# Prevalence of malnutrition based on global leadership initiative in malnutrition criteria for completeness of diagnosis and future risk of malnutrition based on current malnutrition diagnosis: systematic review and meta-analysis

**DOI:** 10.3389/fnut.2023.1174945

**Published:** 2023-07-04

**Authors:** Wentao Bian, Yi Li, Yu Wang, Li Chang, Lei Deng, Yulian Li, Hua Jiang, Ping Zhou

**Affiliations:** ^1^Chengdu University of Traditional Chinese Medicine, Chengdu, China; ^2^Sichuan Provincial People’s Hospital, Chengdu, China; ^3^Institute of Emergency and Disaster Medicine, Provincial People’s Hospital, Chengdu, China

**Keywords:** malnutrition, GLIM criteria, nutritional risk, systematic review, meta-analysis

## Abstract

**Background:**

The proposal of the global leadership initiative in malnutrition (GLIM) criteria has received great attention from clinicians. The criteria are mainly used in the research environment and have the potential to be widely used in the clinic in the future. However, the prevalence of malnutrition and risk of future malnutrition based on a current diagnosis of malnutrition are worth exploring.

**Methods:**

A systematic search of PubMed, Embase, and the Cochrane Library was performed from the earliest available date to 1 February 2023. According to the diagnostic criteria of the GLIM, we analysed the prevalence of malnutrition by directly adopting the GLIM criteria for diagnosis without a previous nutritional risk screening (one-step approach) and by adopting the GLIM criteria for diagnosis after a nutritional risk screening (two-step approach). The main outcome was the prevalence of malnutrition based on the one-and two-step approaches. Secondary outcomes were the future risk of malnutrition based on the GLIM diagnosis, including mortality within and beyond 1 year. primary outcomes were pooled using random-effects models, and secondary outcomes are presented as hazard ratios (HRs) and 95% confidence intervals (CIs).

**Results:**

A total of 64 articles were included in the study, including a total of 47,654 adult hospitalized patients and 15,089 malnourished patients based on the GLIM criteria. Malnutrition was diagnosed by the one-step approach in 18 studies and by the two-step approach in 46 studies. The prevalence of malnutrition diagnosed by the one-and two-step approaches was 53% (95% CI, 42%–64%) and 39% (95% CI, 0.35%–0.43%), respectively. The prevalence of malnutrition diagnosed by the GLIM criteria after a nutritional risk screening was quite different; the prevalence of malnutrition diagnosed by the Nutritional Risk Screening 2002 (NRS2002) GLIM tool was 35% (95% CI, 29%–40%); however, the prevalence of malnutrition diagnosed by the Mini Nutrition Assessment (MNA) GLIM tool was 48% (95% CI, 35%–62%). Among the disease types, the prevalence of malnutrition in cancer patients was 44% (95% CI, 36%–52%), while that in acute and critically ill patients was 44% (95% CI, 33%–56%). The prevalence in patients in internal medicine wards was 40% (95% CI, 34%–45%), while that in patients in surgical wards was 47% (95% CI, 30%–64%). In addition, the mortality risk within 1 year (HR, 2.62; 95% CI, 1.95–3.52; *I*^2^ = 77.1%) and beyond 1 year (HR, 2.04; 95% CI, 1.70–2.45; *I*^2^ = 59.9%) of patients diagnosed with malnutrition by the GLIM criteria was double that of patients with normal nutrition.

**Conclusion:**

The prevalence of malnutrition diagnosed by the GLIM criteria after a nutritional risk screening was significantly lower than the prevalence of malnutrition diagnosed directly by the GLIM criteria. In addition, the mortality risk was significantly greater among malnourished patients assessed by the GLIM criteria.

**Systematic review registration**: identifier CRD42023398454.

## Introduction

Malnutrition is a global problem, and studies have shown that malnutrition can lead to adverse clinical outcomes, including increased morbidity and mortality (1). Previous malnutrition screening tools, such as the Nutritional Risk Screening 2002 (NRS2002) tool, Mini Nutrition Assessment (MNA), and Malnutrition Universal Screening Tool (MUST), can only be used in certain patient populations, and the risk of malnutrition obtained from these screening tools varies widely. The global leadership initiative in malnutrition (GLIM) criteria launched by nutrition experts from various countries in 2018 have been verified to have high diagnostic sensitivity and specificity (2, 3), demonstrating important progress in the diagnosis of malnutrition. Related studies have reported the prevalence of malnutrition in adult hospitalized patients diagnosed by the GLIM criteria, ranging from 19% to 69.7% (4–8).

Currently, the prevalence of malnutrition in adult hospitalized patients based on the GLIM criteria is unclear, and the future malnutrition risk based on a current malnutrition diagnosis is also unknown. This study mainly explored the prevalence of malnutrition based on the GLIM criteria and the future risk of malnutrition based on a current malnutrition diagnosis.

**Figure 1:**
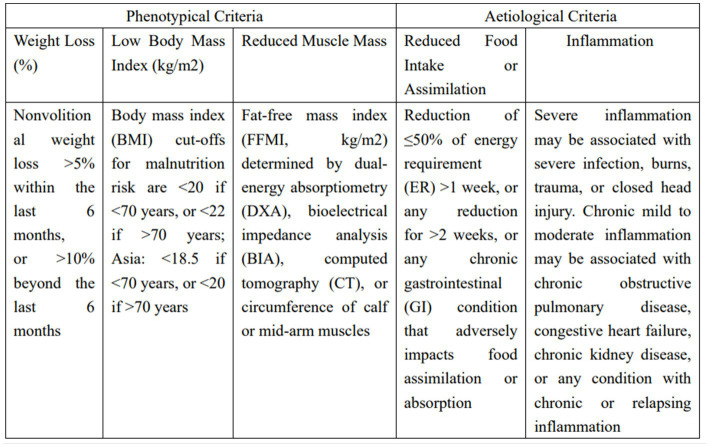
Global leadership initiative in malnutrition (GLIM) phenotypical and aetiological criteria for the diagnosis of malnutrition.

## Methods

### Search strategy

**Figure 2:**
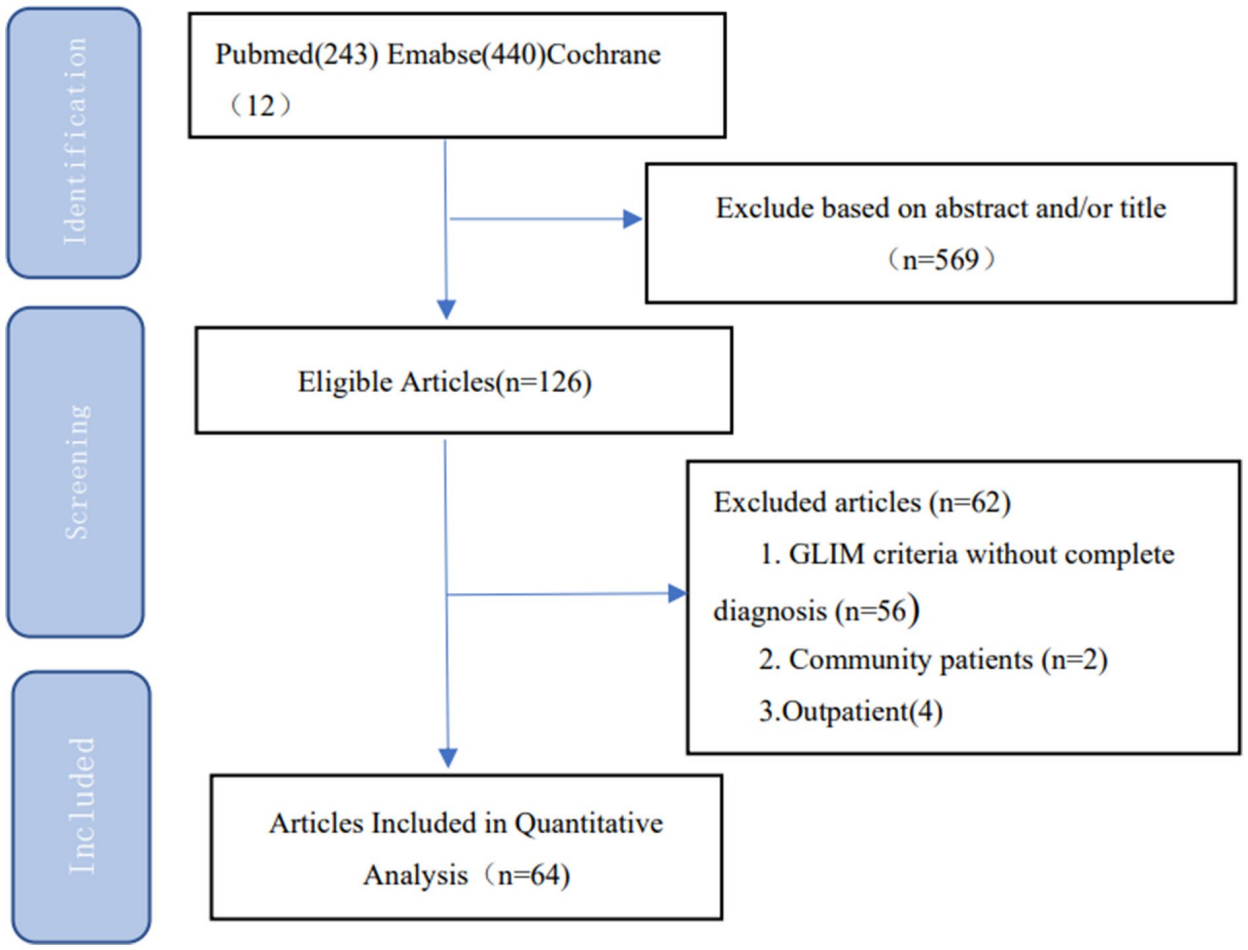
Flow diagram of the search strategy and study selection process.

The meta-analysis was performed according to the Preferred Reporting Items for Systematic Reviews and Meta-Analyses (PRISMA) list, the Guidelines for Meta-Analysis of Observational Epidemiological Studies, and the study protocol (9). We performed a systematic literature search of PubMed, Embase, and the Cochrane Library (up to 10 February 2023) using a combination of MeSH/Emtree terms and title/abstract keywords. The keywords were “malnutrition” and “GLIM criteria”. Supplementary material shows the detailed search strategy. Figure 2 illustrates the complete search strategy. The titles and abstracts of all identified studies were screened by two junior researchers, and articles irrelevant to the research question were excluded. Subsequently, all remaining articles were comprehensively reviewed according to the selection criteria. References were also reviewed to identify other relevant studies. Any discrepancies were negotiated between two senior researchers.

### Inclusion and exclusion criteria

The following inclusion criteria were adopted in this study: (1) the study was a randomized controlled trial, cohort study, case-control study, or cross-sectional study. (2) Based on the complete GLIM diagnostic criteria (Figure 1), there were clear phenotypical and aetiological criteria, including the one-step approach and two-step approach to analyse the prevalence of malnutrition; the one-step approach consisted of directly adopting the GLIM criteria for diagnosis without a previous nutritional risk screening, and the two-step approach consisted of adopting the GLIM criteria for diagnosis after first conducting a nutritional risk screening, with the GLIM criteria for the diagnosis of malnutrition including at least one phenotypical and one aetiological criterion. (3) The research population was hospitalized adults, with no limitations on the type of disease. The exclusion criteria were as follows: (1) the GLIM diagnostic criteria were incomplete, i.e., phenotypical or aetiological criteria were not evaluated or unclear. (2) Patients were nonhospitalized patients, including outpatients, those in nursing homes, and those in other related groups. (3) The study was a review article or case report.

### Data extraction

Two junior researchers independently collected data *via* preestablished forms. Disagreements were resolved through discussion. The collected information mainly included author, year of publication, country or region, one-or two-step approach, initial nutritional risk screening tool, disease type, research design, and nutritional risk (survival and death) of malnourished patients.

### Assessment of study quality

A total of 64 articles were included in the study. We assessed nonrandomized studies using the Newcastle-Ottawa Quality Assessment Scale. Studies were assessed using three categories: study group selection (0–4 points); comparability (0–2 points); and exposure (0–3 points). A total score of ≤3 was considered to indicate low quality; 4–6, medium quality; and ≥7, high quality. These scores were used only to facilitate the interpretation of the meta-analysis results and not as criteria for the inclusion or exclusion of studies. Supplementary Table S2 shows the bias and quality of the included studies.

### Data analysis

The primary objective of this meta-analysis was to investigate the prevalence of malnutrition and the nutritional risk in adult hospitalized patients based on the GLIM criteria. We calculated heterogeneity among studies using Cochran’s *Q* test and the *I*^2^ statistic. All *p*-values were two-tailed, and the results of all analyses were considered statistically significant at *p* ≤ 0.05, except the results of the heterogeneity and publication bias tests. If *I*^2^ > 50%, indicating large heterogeneity among different studies, we applied a random-effects model to calculate the pooled effect value and 95% confidence interval (CI). We also performed subgroup analysis by nutritional diagnostic tool, region and disease type. All statistical analyses were performed using STATA 17.0.

## Results

### Search results

The relevant characteristics of the included literature are shown in Figure 5. There were a total of 64 studies with a total of 47,654 subjects. A total of 18 articles directly adopted the GLIM criteria for diagnosis without a previous nutritional risk screening, and the remaining 46 articles performed a nutritional risk screening first and then followed the GLIM criteria for diagnosis. The nutritional screening tools used included the NRS2002 tool, MUST, MNA, Subjective Global Assessment (SGA), or other risk screening tools and groups of several nutritional screening tools. The types of diseases included cancer, critical illness, COVID-19, stroke, dialysis, and inflammatory bowel disease. Among the 64 articles, 34 were from Asia (10–42), 20 were from Europe (4, 43–61), 9 were from America (62–70), and 1 was from Oceania (71). Of these, 16 studies reported on the future risk of malnutrition diagnosed based on the GLIM criteria (death of malnourished patients in ≤1 year), and 11 studies reported on the future risk of malnutrition diagnosed based on the GLIM criteria (death of malnourished patients in >1 year).

## Outcomes

### Primary outcomes

**Figure 3:**
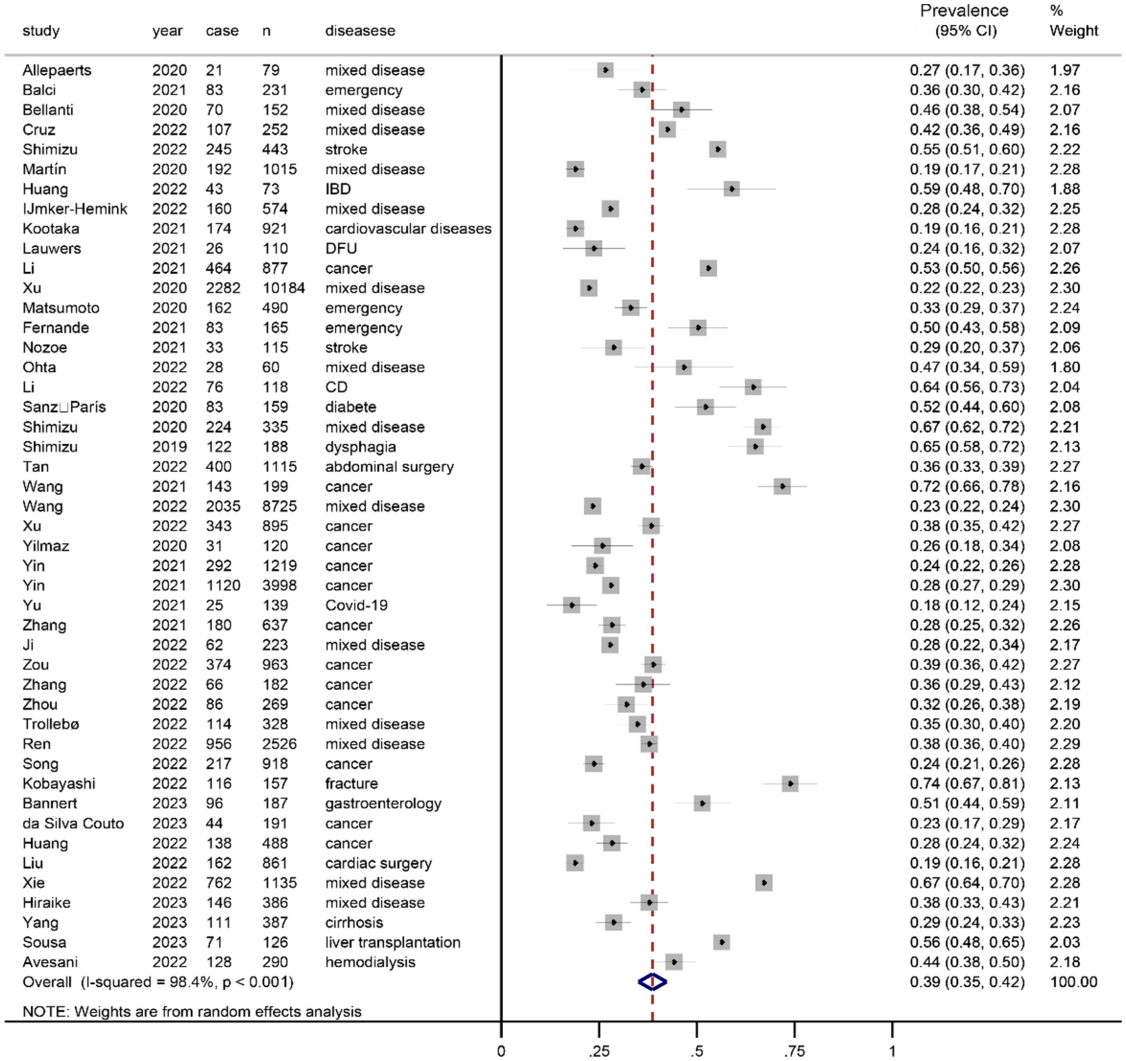
Prevalence of malnutrition diagnosed according to the GLIM criteria after a nutritional risk screening (two-step approach).

**Figure 4:**
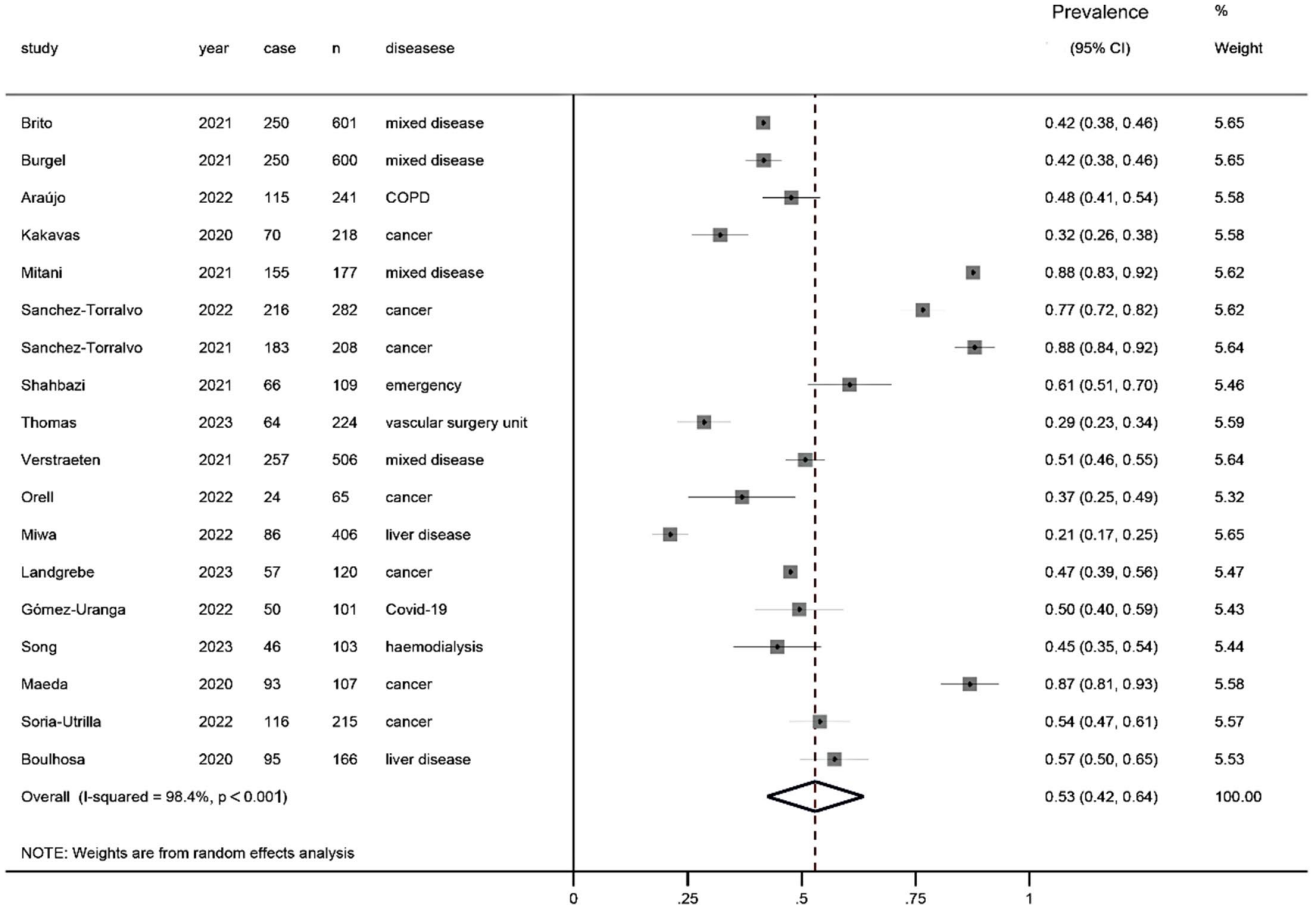
Prevalence of malnutrition diagnosed directly using the GLIM criteria without a nutritional risk screening (one-step approach).

Figures 3 4 show the prevalence of malnutrition based on the GLIM criteria as determined by random-effect model analysis. Figure 4 shows the prevalence of malnutrition based on directly adopting the GLIM criteria for diagnosis (one-step approach), with a summarized prevalence of malnutrition of 53% (95% CI, 42%–64%). Figure 3 shows the prevalence of malnutrition based on the GLIM criteria for diagnosis after first conducting a nutritional risk screening (two-step approach), with a prevalence of 39% (95% CI, 0.35%–0.43%).

**Figure 5:**
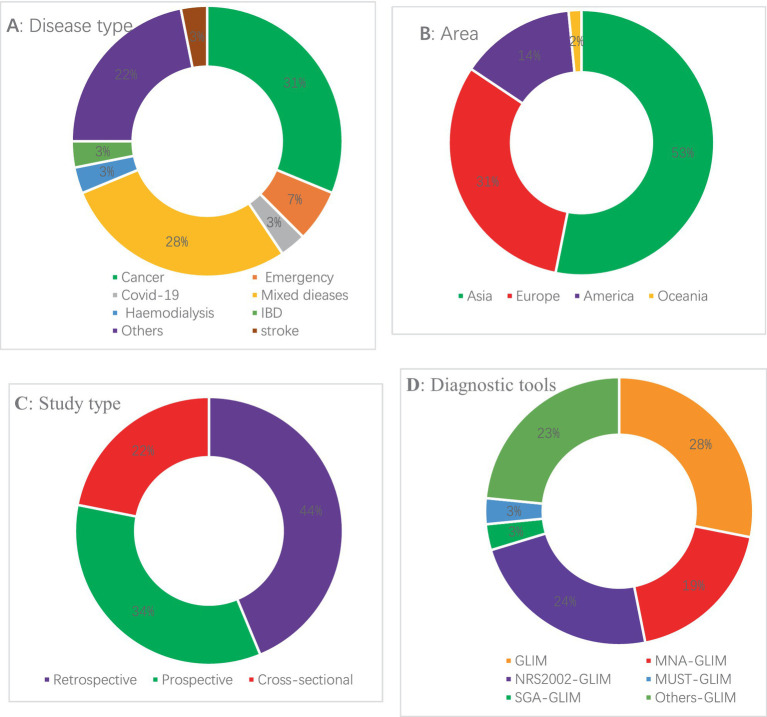
Baseline characteristics of the studies in the meta-analysis (see Supplementary Table S1 for details).

### Secondary outcomes

**Figure 6:**
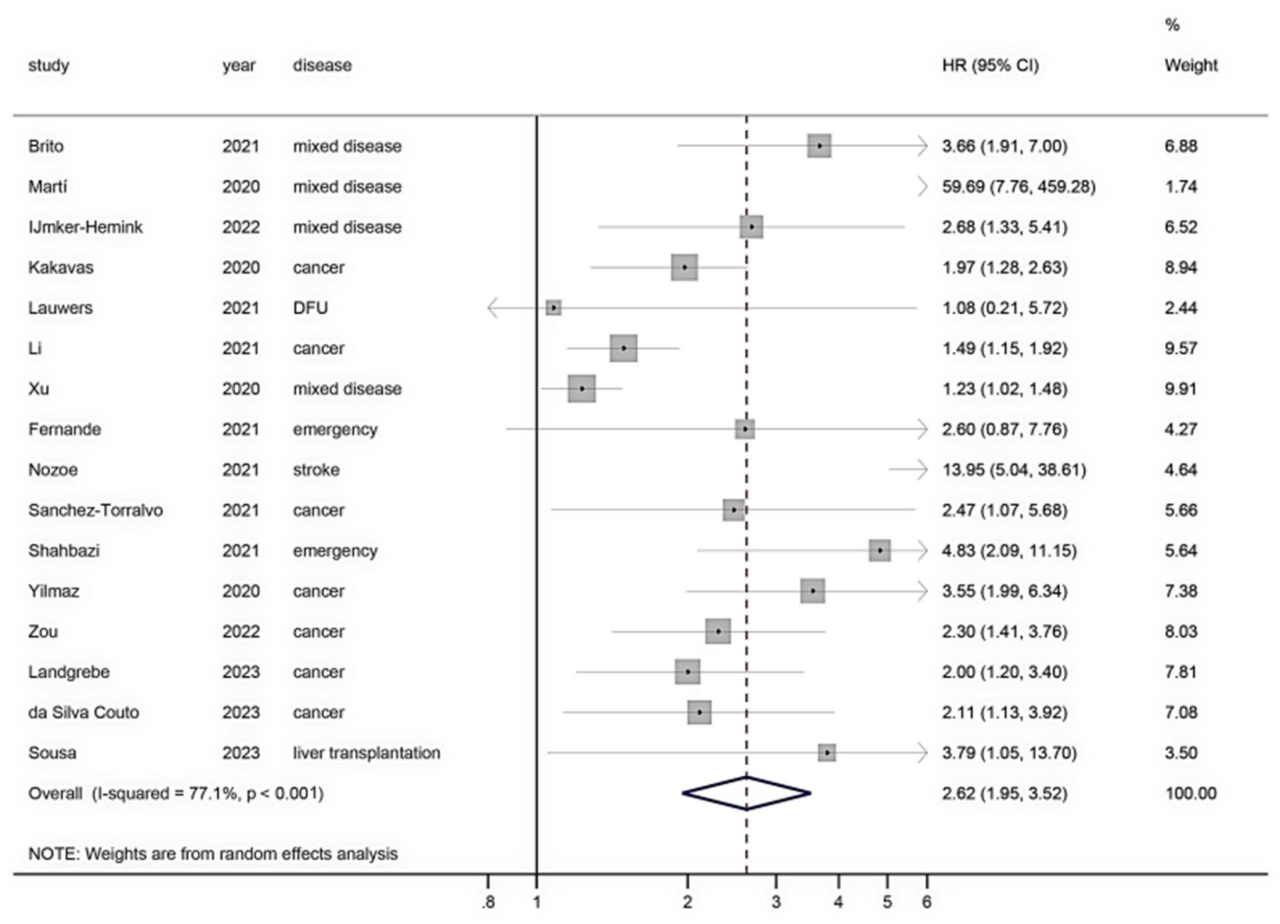
Mortality risk within 1 year of malnourished patients diagnosed by the GLIM criteria.

**Figure 7:**
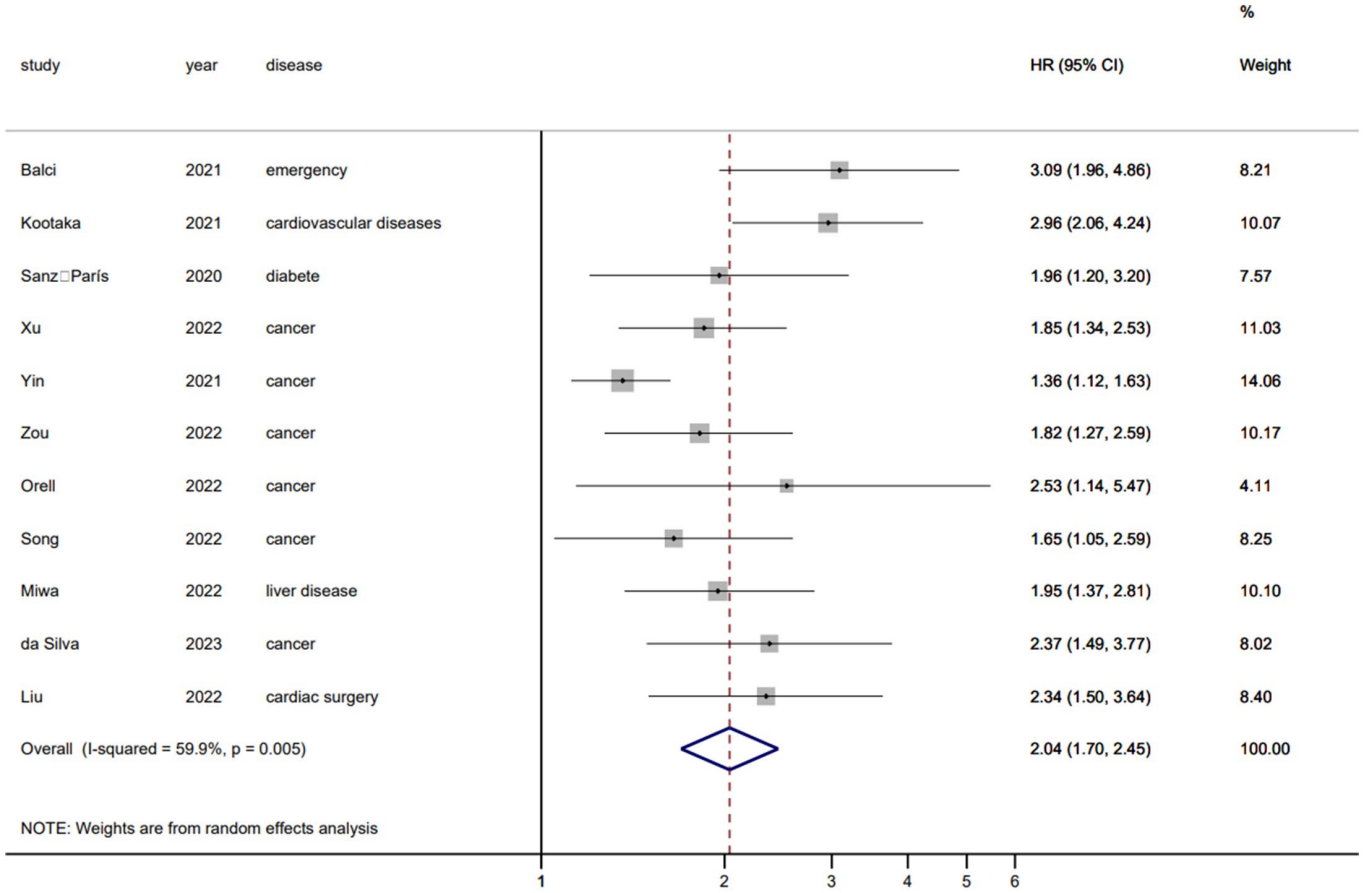
Mortality risk beyond 1 year in malnourished patients diagnosed by the GLIM criteria.

Figure 6 shows the mortality risk of malnourished patients diagnosed based on the GLIM criteria within 1 year (HR, 2.62; 95% CI, 1.95–3.52; *I*^2^ = 77.1%). Figure 7 shows the mortality risk of malnourished patients diagnosed based on the GLIM criteria beyond 1 year (HR, 2.04; 95% CI, 1.70–2.45; *I*^2^ = 59.9%).

### Subgroup analysis

The rates of malnutrition according to different screening tools cannot be compared equally as different tools were used in different cohorts. We simply report the malnutrition risk across cohorts, in each of which a different screening tool was used. As shown in Table 1, the prevalence of malnutrition obtained by the GLIM criteria after a nutritional risk screening was quite different; the prevalence of malnutrition diagnosed by the NRS2002 GLIM tool was 35% (95% CI, 29%–40%); however, the prevalence of malnutrition diagnosed by the MNA GLIM tool was 48% (95% CI, 35%–62%). The prevalence of malnutrition varied little by region. Among the disease types, the prevalence of malnutrition in cancer patients was 44% (95% CI, 36%–52%), while that in acute and critically ill patients was 44% (95% CI, 33%–56%). The prevalence in patients in internal medicine wards was 40% (95% CI, 34%–45%), while that in patients in surgical wards was 47% (95% CI, 30%–64%).

**Table 1:** Relevant subgroup analyses.

Covariates	Subgroup	Number of studies	Prevalence	Heterogeneity *I*^2^
			Random-effects model	
NRS2002 GLIM		15	0.35 (0.29–0.40)	97.4%
MNA GLIM		12	0.48 (0.35–0.62)	99.3%
MUST GLIM		2	0.23 (0.14–0.32)	93.6%
SGA GLIM		2	0.31 (0.16–0.46)	94.9%
Area	Asia	34	0.41 (0.37–0.46)	99.0%
	Europe	20	0.47 (0.37–0.56)	97.8%
	America	9	0.42 (0.32–0.53)	97.3%
Disease type	Oncology	20	0.44 (0.36–0.52)	98.9%
	Emergency	4	0.44 (0.33–0.56)	92.9%
	Internal medicine	26	0.40 (0.34–0.45)	98.7%
	Surgery	5	0.47 (0.30–0.64)	98.8%
	COVID-19	2	0.34 (0.03–0.64)	96.4%

## Discussion

We diagnosed malnutrition as a complete diagnosis based on the GLIM criteria and excluded relevant studies without a complete diagnosis. In fact, the results show that the current diagnosis of malnutrition based on the GLIM criteria is confusing and that most studies do not report a complete diagnosis. Usually, according to the GLIM diagnostic criteria, the diagnosis of malnutrition first requires a nutritional risk screening, followed by a diagnosis according to the GLIM criteria (two-step approach) (3); however, some studies directly used the GLIM criteria to diagnose malnutrition (one-step approach). The prevalence of malnutrition diagnosed by the one-step approach was 53%, while that diagnosed by the two-step approach was 39%. The rate of a positive diagnosis by the one-step approach is higher than that by the two-step approach, possibly because the two-step approach considers not only the symptoms of malnutrition but also the adverse clinical outcomes of nutritional risk (72, 73). Due to the uncertainty of the phenotypical and aetiological components of the GLIM criteria, such as BMI, the criteria for elderly and young individuals are different, and differences may exist among different races (74). In addition, individual studies have used different nutritional risk screening tools and then used the GLIM criteria to obtain different prevalence rates of malnutrition. However, since the tools were not used in the same cohort, it is not yet possible to judge which screening tool is more efficient. For the diagnosis of malnutrition based on the GLIM criteria, a nutritional risk screening is performed first, and different screening tools have been used in different cohorts. The MUST is applicable to all adults but is recommended to be used in community settings (75); the MNA is more suitable for elderly populations (≥65 years old) (76); the NRS2002 tool is applicable to adults aged 18 to 90 years, but its main role is to identify patients with nutritional risk, not to assess the existing nutritional status (77). We pooled the prevalence of malnutrition across the cohorts, and the results showed that the prevalence of malnutrition diagnosed using the MNA GLIM tool was higher than that diagnosed by the NRS2002 GLIM tool, which is consistent with the related research results reported by Xu et al. (17). One reason may be that the MNA GLIM tool is mostly used in elderly patients, and the prevalence of malnutrition due to long-term chronic underlying diseases is high (76). However, the prevalence of malnutrition based on region showed little variation. It is speculated that this result may be due to the body fat content and corresponding BMI cut-off values being higher in European regions than in Asian regions (74, 75). In addition, we analysed the prevalence of malnutrition by disease type, including oncology, emergency, internal medicine, surgery, and COVID-19, and the results showed that the prevalence of malnutrition in surgical wards was slightly higher than that in internal medicine wards. There was also a small difference in the prevalence of malnutrition between oncology and emergency patients, but this analysis was simply based on an aggregation of different cohorts, and the results may be biased.

Involuntary weight loss, which is the most definitive diagnostic indicator of malnutrition, topped the GLIM framers vote. BMI is also a relatively clear quantitative index, and it is easy to obtain and convenient to apply. However, when the GLIM criteria were released, it was also pointed out that there are substantial regional differences in using BMI as a diagnostic standard for malnutrition (3). Reduced body mass index and body composition analysis can objectively and accurately measure body composition. The common methods are the dual energy X-ray absorptiometry method (DEXA) and bioelectrical impedance method. The DXA method mainly uses detectors to detect the absorption of X-rays by the tested parts, thereby calculating body fat composition, non-body fat composition and bone mineral content. These two methods are involved in the literature included in this study. In addition, some studies also use calf circumference for simplified measurement of muscle mass (6, 17). In the burden of disease/inflammation, albumin/prealbumin is one of the commonly used indicators to evaluate liver function, but it is often affected by exogenous infusion, so it cannot accurately evaluate nutritional status, and serum C-reactive protein (CRP) can be compared with good for assessing inflammatory status. Notably, some studies have compared the prevalence of malnutrition between GLIM and ESPEN, and the GLIM criteria have a higher detection rate of malnutrition than the ESPEN criteria (5, 34, 57), possibly because the GLIM criteria add aetiological diagnosis items (3).

In the relevant studies included in this paper, malnourished patients diagnosed according to GLIM criteria may have reported discharge or five-year mortality risk, and we extracted pooled effect values directly from the original papers and pooled them. In addition, in the relevant studies included in this article, most of the mortality rates of malnourished patients diagnosed based on the GLIM criteria were reported using the HR, but a few studies used the odds ratio (OR) to indicate the effect size. Considering that the HR includes the time factor, we partially replaced the ORs reported in the studies with HRs, which may have caused minor bias.

It has been reported that malnutrition diagnosed by the GLIM criteria is significantly associated with a poor prognosis (6, 15, 78); it will not only increase the complications of patients (79) and prolong the length of hospital stay (80) but also increase the mortality rate (73). Our results also showed that malnutrition based on the GLIM criteria was associated with significantly increased mortality within and beyond 1 year, but there was large heterogeneity, which may be due to the use of different screening tools in different patient populations (81). However, although the pooled heterogeneity was large, the pooled effect size was significant. Therefore, the diagnosis of malnutrition by the GLIM criteria can better predict the mortality risk of patients and requires timely nutritional support to improve long-term survival (3). In conclusion, our preliminary findings validate the ability of the “minimum criteria” (at least one phenotypical and one aetiological criterion) for the diagnosis of malnutrition using the GLIM criteria to predict adverse clinical outcomes.

### Limitations

The results of this systematic review and mate-analysis need to be interpreted with caution. First, the GLIM criteria were mainly studied based on a complete diagnosis; although the included articles had clear phenotypical and aetiological criteria, the results may have been biased due to differences in the assessment of BMI and muscle mass loss among studies. Second, regarding the assessment of mortality, most studies reported mortality data using the HR, while a few articles used the OR. We used the HR to indicate the pooled effect size for the articles, which may introduced a small bias. Finally, most articles were from Asia, and these conclusions may not apply to other geographic regions and populations.

## Conclusion

The prevalence of malnutrition diagnosed by the GLIM criteria after a nutritional risk screening was significantly lower than the prevalence of malnutrition diagnosed directly by the GLIM criteria. In addition, the risk of mortality was significantly higher in malnourished patients assessed by the GLIM criteria. In the future, it is necessary to explore the effectiveness of the combination of different nutritional screening tools and the GLIM criteria for the nutritional diagnosis of relevant groups in a large cohort.

## Data availability statement

The original contributions presented in the study are included in the article/Supplementary material, further inquiries can be directed to the corresponding authors.

## Author contributions

WB and PZ contributed to the conception and design of the work. WB, YW, YiL, YuL, and HJ collected information and analyzed data used in the systematic review and meta-analysis. LD polished this article. PZ and HJ substantively revised it. All authors contributed to the article and approved the submitted version.

## Conflict of interest

The authors declare that the research was conducted in the absence of any commercial or financial relationships that could be construed as a potential conflict of interest.

## Publisher’s note

All claims expressed in this article are solely those of the authors and do not necessarily represent those of their affiliated organizations, or those of the publisher, the editors and the reviewers. Any product that may be evaluated in this article, or claim that may be made by its manufacturer, is not guaranteed or endorsed by the publisher.

## Supplementary material

The Supplementary material for this article can be found online at: https://www.frontiersin.org/articles/10.3389/fnut.2023.1174945/full#supplementary-material
