# Supplementary material for: Prevalence of malnutrition based on global leadership initiative in malnutrition criteria for completeness of diagnosis and future risk of malnutrition based on current malnutrition diagnosis: systematic review and meta-analysis
Source: Front Nutr. 2023 Jul 4;10:1174945. doi: 10.3389/fnut.2023.1174945 (PMC10352804; doi:10.3389/fnut.2023.1174945)
Supplement: Supplementary file 1 [file Data_Sheet_1.docx]

**Supplementary Material**

| study | area | No.Malnutrition  (GLIM criteria) | total sample size | Nutritional Risk Screening | Nutritional Risk Screening Tool | Study Type | disease type | Mortality within one year | Mortality over one year |
| --- | --- | --- | --- | --- | --- | --- | --- | --- | --- |
| Allepaerts2020 | Belgium | 21 | 79 | Yes | MNA | Cross-sectional | mixed disease | No | No |
| Balci2021 | Turkey | 83 | 231 | Yes | SGA, NRS-2002 | Cross-sectional | emergency | No | Yes |
| Bellanti2020 | Italy | 70 | 152 | Yes | NRS-2002, SGA,  MUST | Prospective | mixed disease | No | No |
| Boulhosa2020 | Brazil | 95 | 166 | No |  | Prospective | liver disease | No | No |
| Brito2021 | Brazil | 250 | 601 | No |  | Prospective | mixed disease | Yes | No |
| Burgel2021 | Brazil. | 250 | 600 | No |  | Cross-sectional | mixed disease | No | No |
| Cruz2022 | Brazil. | 107 | 252 | Yes | NRS-2002 | Prospective | mixed disease | No | No |
| Araújo2022 | Brazil | 115 | 241 | No |  | Prospective | COPD | No | No |
| Shimizu2022 | Japan | 245 | 443 | Yes | MNA | Cross-sectional | stroke | No | No |
| Martín2020 | Mexico | 192 | 1015 | Yes | NRS-2002 | Prospective | mixed disease | Yes | No |
| Huang2022 | China | 43 | 73 | Yes | NRS-2002 | Retrospective | IBD | No | No |
| IJmker-Hemink2022 | Netherlands | 160 | 574 | Yes | MUST | Retrospective | mixed disease | Yes | No |
| Kakavas2020 | Greece. | 70 | 218 | No |  | Prospective | cancer | Yes | No |
| Kootaka2021 | Japan. | 174 | 921 | Yes | GNRI | Retrospective | cardiovascular diseases | No | Yes |
| Lauwers2021 | Belgium | 26 | 110 | Yes | NRS-2002 | Retrospective | diabetic Foot Ulcer (DFU) | Yes | No |
| Li2021 | China | 464 | 877 | Yes | NRS-2002 | Retrospective | cancer | Yes | No |
| Xu2020 | China | 2282 | 10184 | Yes | NRS2002 or MNA-SF or MUST | Retrospective | mixed disease | Yes | No |
| Matsumoto2020 | Japan. | 162 | 490 | Yes | MNA | Prospective | emergency | No | No |
| Mitani2021 | Japan | 155 | 177 | No |  | Retrospective | mixed disease | No | No |
| Fernande2021 | Brazil. | 83 | 165 | Yes | MNA | Prospective | emergency | Yes | No |
| Nozoe2021 | Japan | 33 | 115 | Yes | MNA | Cross-sectional | stroke | Yes | No |
| Ohta2022 | Switzerland | 28 | 60 | Yes | NRS-2002 or MNA | Prospective | mixed disease | No | No |
| Sanchez-Torralvo, 2022 | Spain. | 216 | 2822 | No |  | Cross-sectional | cancer | No | No |
| Sanchez-Torralvo, 2021 | Spain. | 183 | 208 | No |  | Retrospective | cancer | Yes | No |
| Li2022 | China | 76 | 118 | Yes | NRS 2002 | Retrospective | CD | No | No |
| Sanz‐París2020 | Spain | 83 | 159 | Yes | MNA | Retrospective | diabete | No | Yes |
| Shahbazi2021 | Iran | 66 | 109 | No |  | Prospective | emergency | Yes | No |
| Shimizu2020 | Japan | 224 | 335 | Yes | MNA | Cross-sectional | mixed disease | No | No |
| Shimizu2019 | Japan | 122 | 188 | Yes | MNA | Cross-sectional | dysphagia | No | No |
| Tan2022 | China | 400 | 1115 | Yes | NRS-2002, MUST,SGA | Prospective | abdominal surgery | No | No |
| Thomas2023 | Australia | 64 | 224 | No |  | Retrospective | vascular surgery | No | No |
| Verstraeten2021 | Netherland | 257 | 506 | No |  | Retrospective | mixed disease | No | No |
| Wang2021 | China | 143 | 199 | Yes | MNA,NRS-2002,MUST,PNI | Retrospective | cancer | No | No |
| Wang2022 | China | 2035 | 8725 | Yes | MNA | Cross-sectional | mixed disease | No | No |
| Xu2022 | China | 343 | 895 | Yes | SGA | Retrospective | cancer | No | Yes |
| Yilmaz2020 | Turkey | 31 | 120 | Yes | NRS 2002 | Retrospective | cancer | Yes | No |
| Yin2021 | China | 292 | 1219 | Yes | NRS 2002 | Retrospective | cancer | No | Yes |
| Yin2021 | China | 1120 | 3998 | Yes | NRS 2002 | Retrospective | cancer | No | No |
| Yu2021 | China | 25 | 139 | Yes | NRS 2002 | Retrospective | covid-19 | No | No |
| Zhang2021 | China | 180 | 637 | Yes | NRS2002,MUST,  SGA | Retrospective | cancer | No | No |
| Ji2022 | China | 62 | 223 | Yes | NRS-2002,MNA,  MUST | Prospective | mixed disease | No | No |
| Zou2022 | China | 374 | 963 | Yes | NRS‐2002 | Prospective | cancer | Yes | Yes |
| Zhang2022 | China | 66 | 182 | Yes | NRS-2002 | Cross-sectional | cancer | No | No |
| Zhou2022 | China | 86 | 269 | Yes | NRS-2002,MUST | Retrospective | cancer | No | No |
| Trollebø2022 | Norway | 114 | 328 | Yes | NRS2002 | Prospective | mixed disease | No | No |
| Orell2022 | Finland | 24 | 65 | No |  | Retrospective | cancer | No | Yes |
| Ren2022 | China | 956 | 2526 | Yes | NRS2002 | Retrospective | mixed disease | No | No |
| Song2022 | China | 217 | 918 | Yes | NRS-2002 | Prospective | cancer | No | Yes |
| Kobayashi2022 | Japan | 116 | 157 | Yes | MNA | Retrospective | fracture | No | No |
| Miwa2022 | Japan | 86 | 406 | No |  | Retrospective | liver disease | No | Yes |
| Landgrebe2023 | Germany | 57 | 120 | No |  | Prospective | cancer | Yes | No |
| Bannert2023 | Germany | 96 | 187 | Yes | NRS-2002,MUST | Cross-sectional | gastroenterology | No | No |
| da Silva Couto2023 | Brazil | 44 | 191 | Yes | SGA | Retrospective | cancer | Yes | Yes |
| Huang2022 | China | 138 | 488 | Yes | NRS 2002, GNRI, MNA | Prospective | cancer | No | No |
| Gómez-Uranga2022 | Spain | 50 | 101 | No |  | Cross-sectiona | covid-19 | No | No |
| Liu2022 | China | 162 | 861 | Yes | MUST | Retrospective | cardiac surgery | No | Yes |
| Song2023 | Korea | 46 | 103 | No |  | Retrospective | hemodialysis | No | No |
| Maeda2020 | Spain | 93 | 107 | No |  | Cross-sectional | cancer | No | No |
| Xie2022 | China | 762 | 1135 | Yes | MNA | Cross-sectional | mixed disease | No | No |
| Soria-Utrilla2022 | Spain | 116 | 215 | No |  | Prospective | cancer | No | No |
| Hiraike2023 | Japan | 146 | 386 | Yes | MNA | Prospective | mixed disease | No | No |
| Yang2023 | China | 111 | 387 | Yes | RFH-NPT | Prospective | cirrhosis | No | No |
| Sousa2023 | Brazil | 71 | 126 | Yes | SGA,  RFH-NPT | Prospective | liver transplantation | Yes | No |
| Avesani2022 | Sweden | 128 | 290 | Yes | MIS | Prospective | hemodialysis | No | No |

**Supplementary Table 1.** Baseline characteristics of the studies in the meta-analysis.

| Study | Selection | Comparability | Exposure | Score |
| --- | --- | --- | --- | --- |
| Allepaerts2020 | 2 | 2 | 2 | 6 |
| Balci2021 | 2 | 1 | 2 | 5 |
| Bellanti2020 | 3 | 2 | 2 | 7 |
| Boulhosa2020 | 2 | 2 | 2 | 6 |
| Brito2021 | 3 | 2 | 3 | 8 |
| Burgel2021 | 2 | 2 | 2 | 6 |
| Cruz2022 | 3 | 2 | 2 | 7 |
| Araújo2022 | 2 | 2 | 2 | 6 |
| Shimizu2022 | 2 | 2 | 3 | 7 |
| Martín2020 | 3 | 2 | 2 | 7 |
| Huang2022 | 2 | 2 | 3 | 7 |
| IJmker-Hemink2022 | 2 | 2 | 2 | 6 |
| Kakavas2020 | 2 | 2 | 3 | 7 |
| Kootaka2021 | 3 | 2 | 3 | 8 |
| Lauwers2021 | 1 | 2 | 3 | 6 |
| Li2021 | 2 | 2 | 2 | 6 |
| Xu2020 | 3 | 2 | 2 | 7 |
| Matsumoto2020 | 3 | 2 | 2 | 7 |
| Mitani2021 | 3 | 2 | 3 | 8 |
| Fernande2021 | 3 | 2 | 3 | 8 |
| Nozoe2021 | 3 | 2 | 3 | 8 |
| Ohta2022 | 2 | 2 | 2 | 6 |
| Sanchez-Torralvo, 2022 | 2 | 2 | 2 | 6 |
| Sanchez-Torralvo, 2021 | 2 | 1 | 2 | 5 |
| Li2022 | 2 | 2 | 2 | 6 |
| Sanz‐París2020 | 2 | 2 | 2 | 6 |
| Shahbazi2021 | 3 | 2 | 2 | 7 |
| Shimizu2020 | 2 | 2 | 2 | 6 |
| Shimizu2019 | 3 | 2 | 3 | 8 |
| Tan2022 | 3 | 2 | 3 | 8 |
| Thomas2023 | 3 | 2 | 3 | 8 |
| Verstraeten2021 | 3 | 2 | 3 | 8 |
| Wang2021 | 2 | 2 | 2 | 6 |
| Wang2022 | 3 | 2 | 2 | 7 |
| Xu2022 | 2 | 2 | 3 | 7 |
| Yilmaz2020 | 2 | 2 | 2 | 6 |
| Yin2021 | 2 | 2 | 2 | 6 |
| Yin2021 | 2 | 2 | 3 | 7 |
| Yu2021 | 3 | 2 | 2 | 7 |
| Zhang2021 | 3 | 2 | 3 | 8 |
| Ji2022 | 2 | 2 | 2 | 6 |
| Zou2022 | 1 | 2 | 2 | 5 |
| Zhang2022 | 2 | 2 | 2 | 6 |
| Zhou2022 | 2 | 2 | 2 | 6 |
| Trollebø2022 | 1 | 1 | 2 | 4 |
| Orell2022 | 2 | 2 | 2 | 6 |
| Ren2022 | 2 | 1 | 2 | 5 |
| Song2022 | 2 | 2 | 2 | 6 |
| Kobayashi2022 | 2 | 2 | 2 | 6 |
| Miwa2022 | 3 | 2 | 3 | 8 |
| Landgrebe2023 | 3 | 2 | 3 | 8 |
| Bannert2023 | 2 | 2 | 2 | 6 |
| da Silva Couto2023 | 3 | 2 | 2 | 7 |
| Huang2022 | 2 | 2 | 2 | 6 |
| Gómez-Uranga2022 | 1 | 1 | 2 | 4 |
| Liu2022 | 1 | 1 | 2 | 4 |
| Song2023 | 3 | 2 | 2 | 7 |
| Maeda2020 | 3 | 2 | 3 | 8 |
| Xie2022 | 3 | 2 | 3 | 8 |
| Soria-Utrilla2022 | 1 | 1 | 2 | 4 |
| Hiraike2023 | 3 | 2 | 3 | 8 |
| Yang2023 | 2 | 2 | 2 | 6 |
| Sousa2023 | 2 | 2 | 2 | 6 |
| Avesani2022 | 3 | 2 | 3 | 8 |

**Supplementary Table 2.** Quality of observational studies was assessed with the NEWCASTLE-OTTAWA QUALITY ASSESSMENT SCAL.

**Search strategy:**

**Pubmed**

(((GLIM criteria[Title/Abstract])[Title/Abstract])) AND (((((((Nutritional Deficiency[Title/Abstract])) OR (Nutritional Deficiencies[Title/Abstract])) OR (Undernutrition[Title/Abstract])) OR (Malnourishment[Title/Abstract])) OR (Malnourishments[Title/Abstract])) OR (malnutrition[Title/Abstract]))

**Embase**

Session Results

.......................................................

No. Query Results Results Date

#9. #7 AND #8 440 26 Feb 2023

#8. glim AND criteria 452 26 Feb 2023

#7. #1 OR #2 OR #3 OR #4 OR #5 OR #6 269,574 26 Feb 2023

#6. malnourishments 26 Feb 2023

#5. malnourishment 1,188 26 Feb 2023

#4. undernutrition 12,409 26 Feb 2023

#3. nutritional AND deficiencies 12,044 26 Feb 2023

#2. nutritional AND deficiency 52,916 26 Feb 2023

#1. 'malnutrition'/exp OR malnutrition 221,271 26 Feb 2023

**Cochrane Library**

(GLIM criteria) AND (Nutritional Deficiency) OR (Nutritional Deficiencies) OR (Undernutrition) OR (Malnourishment) OR (Malnourishments) OR (malnutrition)
